# Predator experience changes spider mites’ habitat choice even without current threat

**DOI:** 10.1038/s41598-018-26757-y

**Published:** 2018-05-30

**Authors:** Aoi Murase, Kazuo Fujita

**Affiliations:** 1 Laboratory of Ecological Information, Graduate School of Agriculture, Kyoto University, Kyoto, Japan; 2 Department of Psychology, Graduate School of Letters, Kyoto University, Kyoto, Japan

## Abstract

As recent studies have revealed, previous exposure to a predator can change prey behavior even in the absence of current threat. We hypothesized that experiencing a predator increases prey avoidance of lower-quality resources even in the absence of a predator, which in turn influences the prey’s spatial distribution. We examined these hypotheses using the herbivorous spider mite *Tetranychus kanzawai* and the specialist predatory mite *Neoseiulus womersleyi*. We used *Phaseolus vulgaris* as a high-quality host plant and *Hydrangea macrophylla* as a low-quality host plant. First we examined whether *T. kanzawai* females that were previously exposed to predators preferred *P. vulgaris* to *H. macrophylla* under no current threat more than those without predator experience. Second, we tested the effect of predator experience on dispersal by *T. kanzawai* females on *P. vulgaris* or on *H. macrophylla*. Our results show that: (1) predator-experienced *T. kanzawai* females expressed stronger avoidance of the low-quality plant *H. macrophylla* than those without predator experiences; and (2) *T. kanzawai* females transferred to *H. macrophylla* traveled farther than those on *P. vulgaris*, especially females with previous predator experience. These findings reveal neglected aspects of the evolutionary interaction between predators and the habitat choices of their prey.

## Introduction

An increasing number of studies have found that animals once exposed to predators can reduce potential future threat by changing behavior even before re-encounters with a predator. For example, after an encounter with a parasitoid wasp, water striders change oviposition depth even after the predator is no longer present^1^. Similarly, *Drosophila melanogaster* change their preferred oviposition substrate after a single exposure to parasitoid wasps^2^. Because animals in the wild may not be able to accurately predict future events, such experience-mediated prospective behavioral change may be advantageous. Female spider mites (*Tetranychus kanzawai*) that were initially exposed to predators shift oviposition sites for four days even in the absence of current threat, a response that does not occur when conspecific males were present^3^. These studies and others highlight the multiple ways in which previous exposure to a predator can affect prey behavior in the absence of threat. Such experience-mediated prospective change can be seen in prey dispersal, since emigration-related decisions are based on dispersal costs and benefits^4^. Predator avoidance is one benefit of dispersal^5^; for example, predation risk increases dispersal distance in the spider mite *Tetranychus kanzawai*^6^. It appears that predator exposure may change prey decisions about where to settle, even in the absence of current threat.

Predation pressures can narrow the range of herbivorous arthropods’ host plants^7^; for example, naïve female leaf beetles lay more eggs on the plant, where their eggs can be less predated^8^. Nomikou *et al*. showed that previous exposure to predators decreases the number of whiteflies settling on predator-laden substrates^9^. We hypothesized that exposure to predators make prey more discriminating about quality of food, which induces a stronger avoidance of lower-quality food even in the absence of a predator (Hypothesis 1). Such an experience-mediated prospective change of habitat might change the animals’ spatial distribution via dispersal under no current threat (Hypothesis 2). We examined these two hypotheses using the herbivorous spider mite *Tetranychus kanzawai* and the specialist predatory mite *Neoseiulus womersleyi*^10^, following our recent demonstration of prospective predation avoidance^3^.

## Materials and Methods

### Mites and plants

*Tetranychus kanzawai* is commonly found on wild plants in Japan^11^. They construct three-dimensional webs on leaves, in partial contact with the leaf surface^12^. Females lay eggs on the leaf surface or on the web depending on presence of conspecifics, current predation threat and their previous exposure to predators^3,13–15^. Subjects came from a population collected from kudzu vine (*Pueraria lobate*) in <100-m^2^ area in Kyoto, Japan, in 2014. The mites were maintained on an expanded primary leaf of a bean plant (*Phaseolus vulgaris*) pressed onto water-saturated cotton in a Petri dish (diameter 90 mm, depth 14 mm) (hereafter “leaf dish”). We made 2–3 leaf dishes for mites 6–7 times per week. Each dish was started by introducing 10 mature females and 2 mature males. Five to six leaf dishes were placed in a transparent plastic container and kept in laboratory at 25 °C and 65% RH, with a photoperiod of 16:8 (L:D) h. After 9 days each leaf dish (“9-days leaf dish”) normally contained <20 newly matured females and >100 females in teleiochrisalis phase^16^. To minimize any age effect we used mated females 1 h from maturation (“1-h females”) as subjects. The 30~50 teleiochrysalis females were selected randomly from 9-days leaf dishes, and those females and number-matched matured males from other leaf dishes were transferred together onto a new leaf dish. By controlling relative humidly, we ensured that the females matured simultaneously after 24 h^17^; the newly-matured females and males remained together for 1 h to mate.

*Tetranychus kanzawai* can feed on plants containing toxic secondary chemicals (e.g., *Nerium indicum*^18^ and *Hydrangea macrophylla*^19–21^). *Neoseiulus womersleyi*’s predatory performance can be affected directly and indirectly by their prey species’ host plant^18^. While *T. kanzawai* females can survive and oviposit on *H. macrophylla*, they oviposited fewer eggs and the offspring matured more slowly than on *P. vulgaris* (see Supplementary Information S1). While eggs on *H. macrophylla* were eaten by the predator less often than those on *P. vulgaris*, this difference was unlikely to compensate for reduced oviposition on the former (Supplementary Information S1). On this basis *H. macrophylla* is considered to be a suboptimal host plant. *P. vulgaris* was reared in the laboratory condition. We collected *H. macrophylla* leaves just before preparing experiments. We selected leaves with minimal or no visible damage, and cleaned the leaf surface with a brush. All leaf squares were cut 24–48 h before introduction of *T. kanzawai* females and kept in the laboratory, to minimize possible effects of artificial damage on the mites’ behavior^22,23^.

Predatory mites *N. womersleyi* were collected in Nara, Japan, in 2010, and reared on bean leaf dishes heavily infested with the prey species *T. urticae* (30~50 adult and immature females per leaf). Each randomly selected *N. womersleyi* female was kept in a 1.5 ml micro tube with ca. 0.5-μm water for 2 days (“starved predators”).

### Exp. 1: food choice after exposure to a predator

**Figure 1:**
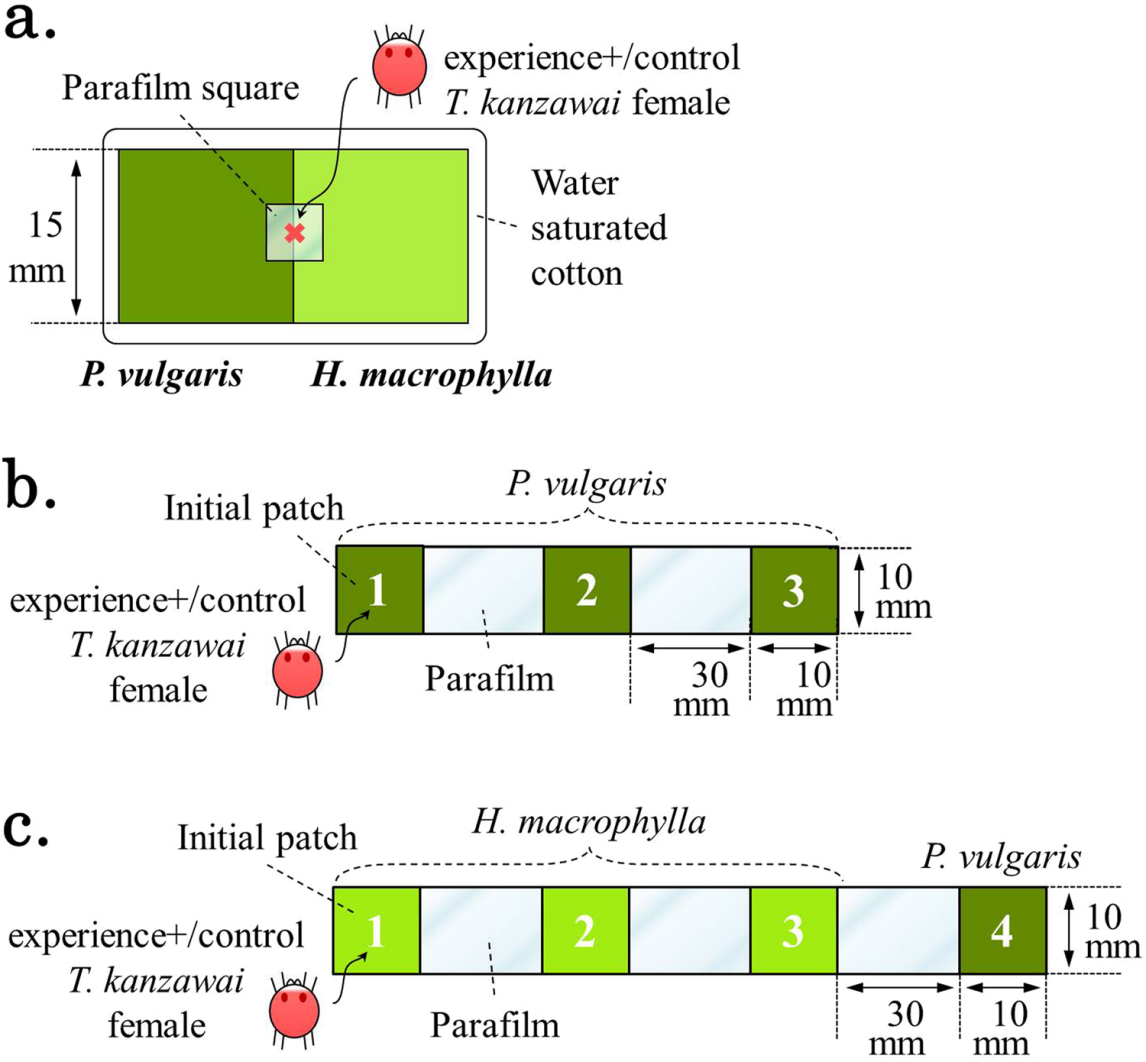
Experimental setups. (**a**) Each control/experience+ female was introduced from the center of a 5 × 5-mm Parafilm square (red cross mark). (**b**) Thirty min after introduction of either a control or experience+ female, the 1^st^ and 2^nd^ patches were connected with 10 × 30-mm Parafilm bridge. (**c**) Patches 1 to 3 were *H. macrophylla*; patch 4 was *P. vulgaris*.

To expose *T. kanzawai* 1-h females to a predator without them being eaten, we introduced each female to a 10 × 10 mm leaf square containing abundant conspecific eggs. Since *N. womersleyi* strongly prefers spider mite eggs to adult females^24^, *T. kanzawai* females experienced predation threat without being eaten or fatally injured. The eggs were laid by three *T. kanzawai* females in the laboratory condition. We removed those females with minimal disturbance to the webs and eggs. To standardize the initial density of eggs we made leaf squares with 25~35 eggs by carefully removing surplus eggs using a brush. Fifteen min after introduction of 1-h females, the predator was introduced onto half of the leaf squares from one corner of the square (experience+). On the other half of the leaf squares, 1-h females cohabited only with conspecific eggs (control). After 24 h in the laboratory condition, all experience+ (n = 17) and control (n = 17) *T. kanzawai* females were introduced onto 5 × 5 mm squares of Parafilm (Parafilm M; American National Can Co., Chicago, IL, USA), and these were placed over the border of 15 × 15 mm *P. vulgaris* and *H. macrophylla* leaf squares in contact with each other on water-saturated cotton (Fig. 1a); the females could easily move between the two plant species. The females always moved onto one of the paired leaf squares within 5 min, after which we removed the Parafilm squares. The setups were maintained in transparent plastic containers in the laboratory for 2 days. We recorded the leaf on which the females had settled by the presence of webs and eggs each day.

### Exp. 2: change in distribution after experience

To examine the effect of experience-mediated avoidance of low-quality food shown in Exp. 1 on dispersal behavior, we connected: (1) three 10 × 10 mm leaf squares (Fig. 1b); and (2) three 10 × 10 mm *H. macrophylla* leaf squares and one 10 × 10 mm *P. vulgaris* leaf square (i.e. the last patch) (Fig. 1c), in line with 10 × 30 mm Parafilm bridges. All setups were put on water saturated cotton, which prevented the females from escaping. We prepared experience+ and control females as in Exp. 1. We introduced experience+ or control females on the center of the initial patch on each setup using a brush. The initial patch was connected to other leaf squares only after a 30-min post-introduction acclimation. Since *T. kanzawai* females appear not to detect plants more than 30 mm away^6^, we assume that dispersing spider mites abandon the previous patch without being attracted to adjacent patches. We used three leaf squares because *T. kanzawai* females reportedly traveled 2.50 ± 0.56 (average ± SE) patches from the initial leaf square when a predator was on the patch (Sakurada & Yano, unpublished). We used *P. vulgaris*, the more attractive plant, at the end of the setups of *H. macrophylla* (Fig. 1c): (1) to trap individuals reaching the end of the experimental environment; and (2) to imitate the mixed vegetation in the wild. All setups were maintained in transparent plastic containers in the laboratory for 24 h, and we determined the leaf on which the females had settled by the presence of webs and eggs.

### Data analysis

We analyzed the proportion of females settled on *H. macrophylla* (Exp. 1) with GLM binomial test (link = logit). In Exp. 2 we used GLM binomial test considering experience of predation risk (experience+, control) and plant species (*P. vulgaris*, *H. macrophylla*) as main factors and their interaction to examine how these factors influence dispersal behavior of *T. kanzawai*. Because we found a significant interaction between experience and plant (see Results), we performed pairwise comparisons using the GLM binomial test and pairwise comparisons by the Bonferroni method based on a significance level of α = 0.05. The proportion of females transferred onto *H. macrophylla* reaching *P. vulgaris* (i.e. 4^th^ patch) was analyzed with GLM binomial test (link = logit). In Exp. 2, we excluded one experience+ female observed on the 4^th^ patch because it neither constructed webs nor laid eggs (see Dataset S2). The analyses were done using free software “R i386 3.4.4” and “JMP 12.1”.

## Results

**Figure 2:**
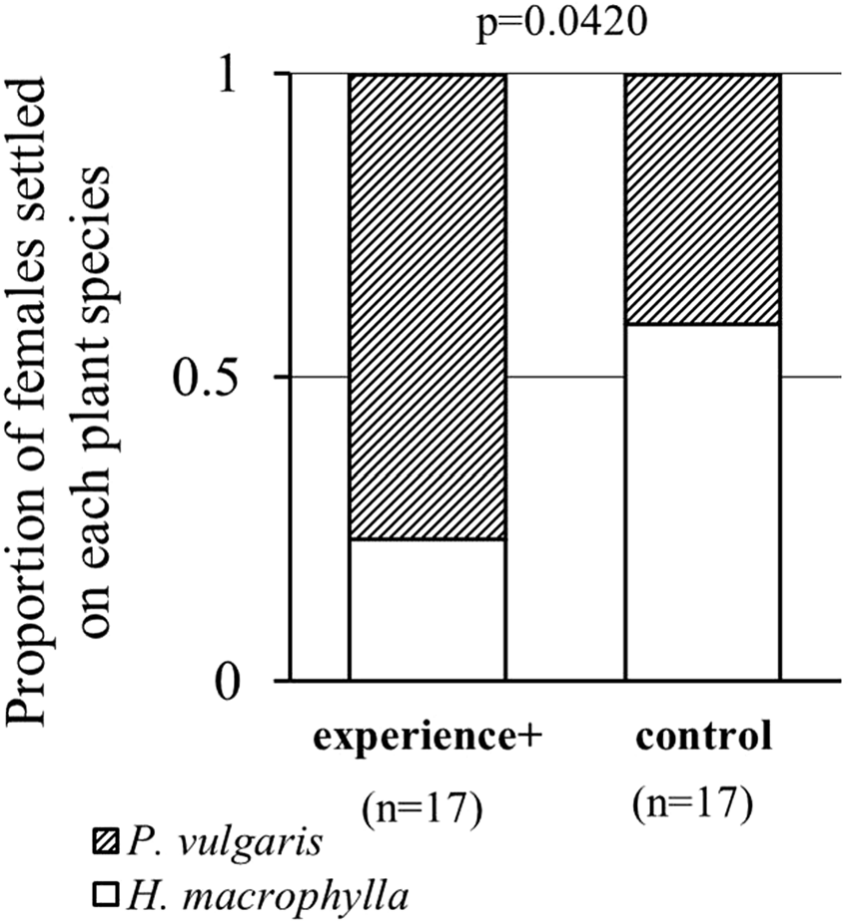
Effect of predator experience on host plant evaluation between *P. vulgaris* and *H. macrophylla* by *T. kanzawai* females. The proportion of females settled on *H. macrophylla* in experience+ subjects (n = 17) was significantly lower than in controls (n = 17) (p = 0.042; GLM, binomial).

In Exp. 1, we examined whether previous exposure to predators would make *Tetranychus kanzawai* females avoid settling on lower-quality food in the absence of a predator. Twenty-four h after the introduction, 10 of 17 control females were settled on *H. macrophylla*, compared to only 4 of 17 experience+ females (p = 0.042; GLM, binomial, link = logit) (Fig. 2). On Day 2, no new webs on the alternative leaf square were built (i.e., no female moved between the leaves).

**Figure 3:**
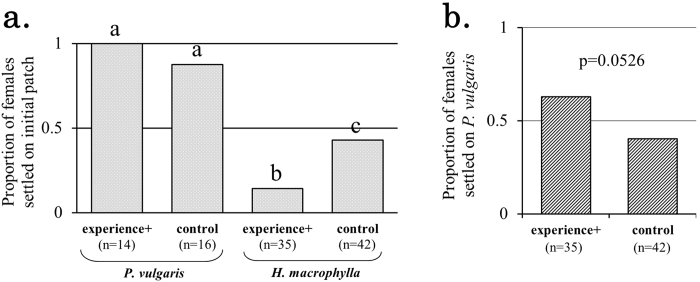
Effect of predator experience on dispersal on *P. vulgaris* and *H. macrophylla* by *T. kanzawai* females. (**a**) The letters a, b and c show the significant differences in the proportion of females settled on the initial patch between four treatments (i.e., experience +/control × *P. vulgaris*/*H. macrophylla*) (GLM binomial pairwise test; Bonferroni adjustment). (**b**) The experience+ females (n = 35) on *H. macrophylla* tended to reach the 4^th^ *P. vulgaris* patch more than controls (n = 42) (p = 0.0526; GLM binomial).

In Exp. 2, we examined the effect of experienced-mediated avoidance of low-quality food shown in Exp. 1 on dispersal behavior. Twenty-four h after introduction onto *P. vulgaris* 14 of 16 control females and all 14 experience+ females remained on the initial patch. By contrast, 24 h after introduction onto *H. macrophylla* the number of individuals settled on the 1^st^ to 4^th^ patches was 18, 4, 3, 17 for control females (n = 42) and 5, 7, 1, 22 for experience+ females (n = 35), respectively. GLM binomial test of the proportion of females settled on the initial patch showed a significant host-plant effect (χ^2^_1_ = 44.90, p < 0.0001) and an interaction between experience and host plant (χ^2^_1_ = 5.76, p = 0.0164) (Fig. 3a). Following the GLM binomial pairwise tests, a significant difference was detected between “experience+ on *H. macrophylla*” and “control on *H. macrophylla*” (p = 0.005). The proportion of females settled on the initial patch in both experience+ and control females was not significantly different from zero (p = 1.0 in experience+, p = 0.995 in control; GLM binomial). The proportion of females on *H. macrophylla* reaching the 4^th^ patch (*P. vulgaris*) tended to be higher in experience+ than control females (p = 0.0526; GLM binomial) (Fig. 3b).

## Discussion

In Exp. 1, we observed the effect of previous exposure to a predator in *T. kanzawai* on food choice between high- and low-quality plants in the absence of current threat (Fig. 1a). The results showed that exposure to a predator clearly induced avoidance of *H. macrophylla* in *T. kanzawai* females (Fig. 2), in support of Hypothesis 1 (see Introduction). Exposure might have enhanced general sensitivity, thereby improving detection of toxic chemicals associated with *H. macrophylla*^20,21^. The discovery of predation risk might have increased risk aversion and hence avoidance of the cost of settlement on *H. macrophylla* (see Supplementary Information S1). Although studies of food aversion in arthropods show that food and aversive substances are often associated e.g.^25^, our results suggest dissociation of the odor of *P. vulgaris* and stimuli from a predator; otherwise experience+ females should have avoided *P. vulgaris*, resulting in settlement on *H. macrophylla*. The absence of such association between a nutritious host plant and predator stimuli in *T. kanzawai* may be adaptive for reproductive efficiency after threat experience.

Exp. 2 was designed to examine the effect of experience-mediated avoidance of low-quality food shown in Exp. 1 on dispersal behavior in *T. kanzawai* (Fig. 1b,c). The significant differences in the proportion of females settled on the initial patch between four treatments in Exp. 2 (Fig. 3a) suggest that: (1) regardless of predator experience, females on *P. vulgaris* seldom abandoned the initial patch; (2) when *T. kanzawai* female were transferred on *H. macrophylla*, they abandoned the initial patch more than those on *P. vulgaris* regardless of their predator experiences, possibly because *H. macrohpylla* is nutritiously dissatisfying for *T. kanzawai*; and (3) females on *H. macrophylla* abandoned the initial patch significantly more often when they had the predator experience. These results support our hypothesis that predator experience can change prey’s spatial distribution even in the absence of current threat (Hypothesis 2; see Introduction).

Interestingly, predator-induced stronger aversion to *H. macrophylla* tended to increase the chance of experience+ *T. kanzawai* females finding the *P. vulgaris* patch, the high-quality plant (Fig. 3b). In other words, even though *T. kanzawai* females should have been uncertain of finding another habitat after abandoning the initial patch^6^, experience+ females decided to travel farther, resulting in reaching a more desirable habitat. If they have eaten insufficiently during exposure to a predator, leading to raised nutritional requirements, they should have stayed on the initial patch (i.e. compromised with *H. macrophylla*) rather than abandoning the initial patch. We consider that the avoidance of *H. macrophylla* and the greater dispersion on *H. macrophylla* by experience+ females might reflect the experience-mediated strategy for more efficient reproduction by finding a high-quality host plant. The experience-mediated prospective change in the females’ decision about where to settle may lead *T kanzawai* females to greater success in finding nutritiously rich habitats in the wild.

The effects of predator experience on habitat choice under no current threat could be assessed in many species, with implications for the evolutionary significance of such prospective changes in spatial distribution of prey species. Considering that plant-specific selection pressure shapes the population^26^, and populations of *T. kanzawai* often develop reproductive incompatibility with other populations from different host plants^27^, we can assume that the experience-mediated change of spatial distribution in *T. kanzawai* females may have implications for speciation. Our study therefore raises a new question: can a single threat experience potentially become a suppressing or facilitating factor in speciation? Further studies might reveal an evolutionary role of learning and its consequences for facilitating or suppressing speciation in animals.

### Data accessibility

All datasets are available in the electronic supplementary material (Datasets S1 to S2).

### Ethical Statements

We declare that all mites and plants were not endangered species, and ethical approval was not required. All of experimental subjects were collected under the permission of Graduate School of Agriculture, Kyoto University.

## Electronic supplementary material

Supplementary Information S1

Dataset S1

Dataset S2
