# Supplementary material for: Predator experience changes spider mites’ habitat choice even without current threat
Source: Sci Rep. 2018 May 30;8:8388. doi: 10.1038/s41598-018-26757-y (PMC5976667; doi:10.1038/s41598-018-26757-y)
Supplement: Supplementary file 1 — Supplementary Information S1 [file 41598_2018_26757_MOESM1_ESM.docx]

**Supplementary Information S1**

**Predator experience changes spider mites’ habitat choice even without current threat.**

Aoi Murase^1*^, Kazuo Fujita^2^

^1^Laboratory of Ecological Information, Graduate School of Agriculture, Kyoto University, Kyoto, Japan

^2^Department of Psychology, Graduate School of Letters, Kyoto University, Kyoto, Japan

^*^Corresponding author

[murase.aoi.45v@ kyoto-u.ac.jp](mailto:murase.aoi.45v@st.kyoto-u.ac.jp)

**Oviposition and maturation following transfer to *H. macrophylla* in *T. kanzawai***

Experimental subjects came from the same population of *Tetranychus kanzawai* maintained on *Phaseolus vulgaris* (common bean) in the laboratory since 2014. We first examined survivorship in the 2014 population of *T. kanzawai* on *Hydrangella macrophilla*, and the consequence of host transfer by testing oviposition and maturation in a forced-choice situation.

We counted the number of eggs laid by each mated *T. kanzawai* female 0-24 h after maturation (0-24h females) for 2 days on 15x15-mm leaf square of *P. vulgaris* (n=22) or *H. macrophylla* (n=21). Each 15x15-mm leaf square of *P. vulgaris* or *H. macrophilla* was on water-saturated cotton in petri dishes, to prevent *T. kanzawai* from escaping*.* After 2 days of oviposition on each leaf square at 25°C and 65% RH, with a photoperiod of 16:8 (L:D) h (hereafter “the laboratory condition”), we removed all females, with minimal disturbance using a fine brush. The eggs were hatched simultaneously by controlling relative humidity. From the next day onward, the number of matured individuals on each leaf was counted daily. We calculated the average maturation rate and days until maturation on each leaf square of *P. vulgaris* or *H. macrophylla*. All leaf squares were kept in transparent plastic containers in the laboratory condition.

All females on *P. vulgaris* and *H. macrophylla* oviposited. The average numbers of eggs per female for 2 days (±SE) were 19.82±0.60 on *P. vulgaris* and 11.76±0.48 on *H. macrophylla* (p<0.001; GLM, Poisson, link=log) (Fig S1a). The average maturation rate of offspring (±SE) was 0.90±0.018 on *P. vulgaris* (n=22 including 390 mites) and 0.92±0.032 on *H. macrophylla* (n=21 including 226 mites) (p=0.79; GLM, binomial, link=logit) (Fig S1b). Average latency until maturation (±SE) was 5.69±0.097 days on *P. vulgaris* and 6.72±0.085 days on *H. macrophylla* (p<0.001; GLM, Gamma, link=inverse) (Fig S1c). These results suggested that *H. macrophylla* could be a potential host plant for *T. kanzawai* but at the cost of reduced oviposition and longer latency to maturation.

**
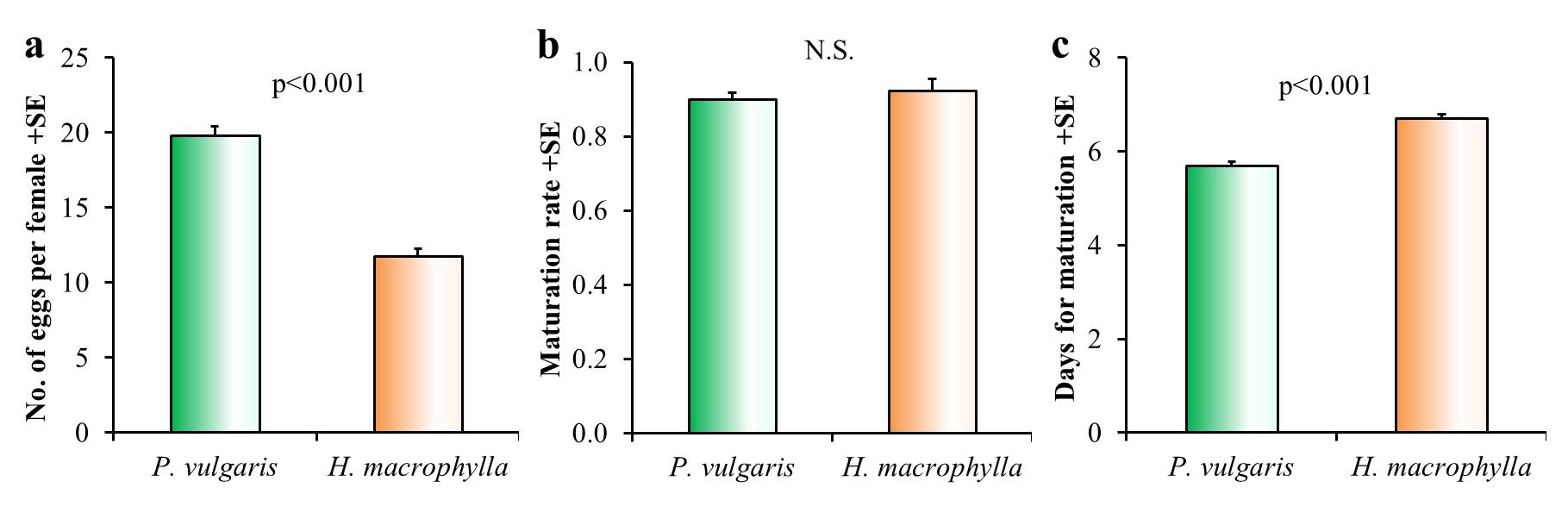
**

**Fig S1. Forced-choice test by *T. kanzawai* on *P. vulgaris* or *H. macrophylla*.**

**a.** Females on *P. vulgaris* (n=22) laid significantly more eggs than those on *H. macrophylla* (n=21) (GLM, Poisson). **b.** Maturation rate of *T. kanzawai* offspring was not significantly different between *P. vulgaris* and *H. macrophylla* groups (GLM, binomial). **c.** *T. kanzawai* offspring on *P. vulgaris* matured significantly faster than those on *H. macrophylla* (GLM, Gamma).

**Egg predation by predatory mites *N. womersleyi* on *H. macrophylla***

Whereas transfer to a new plant under no predation risk is costly for oviposition and maturation, it can be beneficial under predation risk [1, 2]. Given that many studies have shown that predation is an important selection pressure for herbivores on plant hosts [e.g. 3], the cost and benefit of transferring to a novel plant in the presence of predators should be taken into account when measuring the fitness consequences of host transfer. Therefore, we examined whether *T. kanzawai* eggs on *H. macrophylla* would be preyed upon less than those on *P. vulgaris* by the specialist predatory mite, *Neoseiulus womersleyi*. First we introduced one 0-24h *T. kanzawai* female onto each 15x15-mm leaf square of *P. vulgaris* (n=23) or *H. macrophilla* (n=18)*.* Two days after introduction, we removed the females, with minimal disturbance by using a brush. We then introduced a *N. womersleyi* onto each leaf square. To standardize starvation of *N. womersleyi* females, each randomly selected female was kept in a 1.5-mL micro tube with ca. 0.5-μm water for 2 days. We put the leaf squares on water-saturated cotton in petri dishes to prevent starved predators from escaping. After introduction of a predator, all leaf squares were kept for 18 h in transparent plastic containers at 25°C and 65% RH, with a photoperiod of 9:8:1 (L:D:L) h. We then removed all predators and counted the number of surviving eggs.

The initial average number of eggs (±SE) on *P. vulgaris* : *H. macrophylla* was 20.70±0.64 : 11.11±0.54. Eighteen h after introduction of the predator, the average number of predated eggs (±SE) on *P. vulgaris* : *H. macrophylla* was 8.04±0.28 : 5.78±0.38 (p=0.00695; GLM, Poisson, link=log) (Fig. S2a), whereas that of surviving eggs (±SE) on *P. vulgaris* : *H. macrophylla* was 12.65±0.69 : 5.33±0.55 (p<0.001; GLM, Poisson, link=log) (Fig. S2b).

Additionally to examine whether the difference in egg density might affect predation performance by *N. womersleyi*, we prepared 15x15-mm leaf squares of *P. vulgaris* on which we allowed 0-24h *T. kanzawai* females to oviposit for 1 day (hereafter “1d-leaf”; n=16) or for 2 days (“2d-leaf”; n=18). The initial average number of eggs (±SE) on 1d-leaf : 2d-leaf was 11.81±0.55 : 21.39±0.89. We introduced a starved *N. womersleyi* onto the leaf squares in the same way as previously. Eighteen h after introduction of introduction of the predator, the average number of predated eggs (±SE) on 1d-leaf : 2d-leaf was 8.13±0.29 : 7.50±0.34 (p=0.515; GLM, Poisson, link=log).

Thus, the significant difference in the number of predated eggs on *P. vulgaris* compared to *H. macrophylla* is not due to difference in initial egg density. It is known that *N. womersleyi*’s predatory performance can be affected by their prey species’ host plant. For example, moisture from a host plant rich in secondary compounds and feeding on mites acclimated to that plant reduces egg production in *N. womersleyi* [4, 5]. Conceivably, *N. womersleyi* predated fewer eggs on *H. macrophylla* to avoid negative effects of ingesting secondary chemicals [6, 7]. Regardless of mechanisms, our result shows that *T. kanzawai* eggs on *H. macrophylla* have the advantage of reduced predation by *N. womersleyi*. Considering that more eggs survived on *P. vulgaris* than on *H. macrophylla*, however, the advantage of predation avoidance on *H. macrophylla* is unlikely to compensate for the reduced oviposition.


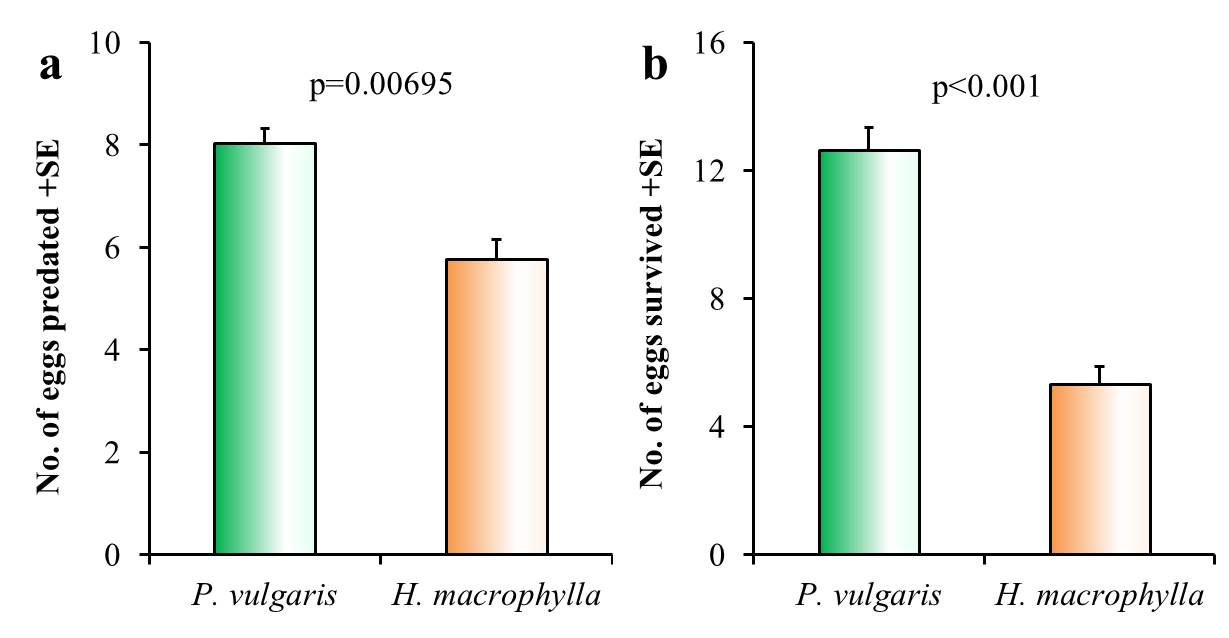


**Fig S2. No-choice test by *N. womersleyi* females on *P. vulgaris* or on *H. macrophylla*.**

**a.** The average number of predated eggs after 18 h was significantly lower on *H. macrophylla* (n=18) than on *P. vulgaris* (n=23) (p=0.007, GLM, Poisson). **b.** The average number of surviving eggs after 18 h was significantly higher on *P. vulgaris* than on *H. macrophylla* (p<0.001, GLM, Poisson).

**References**

1. Ballabeni, P. & Rahier, M.A. quantitative genetic analysis of leaf beetle larval performance on two natural hosts: including a mixed diet. *J. Evol. Biol.* **13**, 98-106 (2000). (doi:10.1046/j.1420-9101.2000.00144.x)
2. Ballabeni, P., Wlodarczyk, M. & Rahier, M. Does enemy-free space for eggs contribute to a leaf beetle’s oviposition preference for a nutritionally inferior host plant? *Funct. Exol.* **15**, 318-324 (2001). (doi:10.1046/j.1365-2435.2001.00529.x)
3. Bernays, E.A. Host range in phytophagous insects: the potential role of generalist predators. *Evol. Ecol*. **3**, 299-311 (1989). (doi: 10.1007/BF02285261)
4. Tajima, R. Specific adaptation of sympatric populations of the Kanzawa spider mite, *Tetranychus kanzawai* (Acari : Tetranychidae) to three host plants. *J. Acarol. Soc. Japan.* **16**, 211-27 (2007).
5. Suzuki, H. et al. Kanzawa spider mites acquire enemy-free space on a detrimental host plant, oleander. *Entomol. Exp. Appl.* **138**, 212–222 (2011). (doi: 10.1111/j.1570-7458.2010.01092.x)
6. Asahina, Y. & Miyake, K. Hydrangenol, a chemical constituent of *Hydrangea hortensia*. *J. Pharmaceut. Soc. Jap.* **408**, 121-126 (1916).
7. Asen, S., Cathey, H.M. & Stuart, N.W. Enhancement of gibberellin growth-promoting activity by hydrangenol isolated from leaves of *Hydrangea macrophylla*. *Plant. Physiol*. **35(6)**, 816-819 (1960).
